# Metabolomics and physiological analysis of the effect of calcium supplements on reducing bone loss in ovariectomized rats by increasing estradiol levels

**DOI:** 10.1186/s12986-021-00602-y

**Published:** 2021-07-23

**Authors:** Hongmei Mao, Wenjun Wang, Lili Shi, Chen Chen, Chao Han, Jinpeng Zhao, Qin Zhuo, Shi Shen, Yan Li, Junsheng Huo

**Affiliations:** 1 National Institute for Nutrition and Health, Chinese Center for Disease Control and Prevention, 29 Nanwei Road, Beijing, 100050 China; 2 Institute of Food Science and Technology, Chinese Academy of Agricultural Sciences, Beijing, 100193 China

**Keywords:** Calcium, Estradiol, Bone mineral density, Osteoporosis, Metabolomics

## Abstract

**Background:**

Data from the 2010–2012 Chinese National Nutrition and Health Survey showed that the vast majority of postmenopausal women in China had dual deficiencies in calcium and estrogen.

**Objective:**

This study aimed to clarify whether calcium supplementation alleviated bone loss caused by calcium restriction combined with estrogen deficiency in rats.

**Methods:**

Forty-eight female rats aged 9 weeks were assigned to 4 groups and fed a low-calcium diet: sham-operated (SHAM-LC), ovariectomized (OVX-LC), and ovariectomized rats treated with 750 mg/kg (OVX-LC-M) or 2800 mg/kg CaCO_3_ (OVX-LC-H). CaCO_3_ or distilled water was administered orally for 13 weeks. Bone mineral density (BMD) and histomorphometry of the femur, serum biochemical parameters, and serum metabolites were analyzed.

**Results:**

The OVX-LC rats showed a significant increase in body weight and serum levels of lipid markers, a significant decrease in serum estradiol, calcium, phosphorus, and 25(OH)D levels, and deterioration of the femur. At 750 mg/kg and 2800 mg/kg, CaCO_3_ reduced the deterioration of trabecular bone and increased the trabecular area percentage (Tb.Ar %) and BMD of the femur. Serum estradiol levels increased in a dose-dependent manner after CaCO_3_ supplementation (*p* < 0.01). The administration of 2800 mg/kg CaCO_3_ decreased serum triglyceride and high-density lipoprotein levels (*p* < 0.05) and decreased the levels of the bone turnover markers osteocalcin, N-telopeptide of type I collagen and β-crosslaps. The results of the metabolomics analysis showed that the glycerophospholipid metabolism pathway was closely related to calcium supplementation, and more DG (44:6 n3), LysoPC (22:2) and PE (P-34:3) and less Cer (d43:0) and PE-NMe2 (46:3) were produced.

**Conclusions:**

The results clearly indicated that calcium supplementation was beneficial for decreasing bone loss in OVX-LC rats. The present study is the first to show that calcium supplementation increased the estradiol content in OVX-LC rats, and the effect of calcium on bone loss may be partially attributed to the increase in the estrogen level that subsequently induced the changes in metabolite levels, eventually increasing the bone mineral density to a relatively higher level to reduce bone deterioration.

## Introduction

Osteoporosis is a disease characterized by a decrease in bone mineral density and the destruction of the bone microstructure, which eventually leads to increased bone fragility and fracture [1–3]. With the aging of the population, osteoporosis has become a common disease that endangers the health of elderly individuals worldwide and has imposed heavy economic and social burdens [4]. According to an epidemiological survey of osteoporosis in China in 2018, the incidence of osteoporosis in people over 50 years old was 19.2%, and the incidence was even higher at 30% and 50% for females over 50 and 65 years old, respectively [5].

Many factors contribute to osteoporosis, such as the insufficient intake of calcium (Ca) and protein, a low level of vitamin D, endocrine factors, heredity, and living habits [6–11]. For postmenopausal women, osteoporosis is mainly caused by estrogen deficiency. The 2010–2012 Chinese National Nutrition and Health Survey showed that the daily Ca intake of Chinese residents was 366.1 mg/d, which was only half of the recommended Ca intake, and the Ca intake of 96.6% of Chinese residents was lower than the estimated average requirement for Ca [12]. Therefore, the vast majority of postmenopausal women in China are present with dual deficiencies in Ca and estrogen.

Ca is an important component of bone, and > 99% of Ca in the body is deposited in bone and teeth [13]. Ca supplementation has been widely used to prevent osteoporosis and subsequent fractures in postmenopausal women based on the hypothesis that an adequate Ca level is crucial for maintaining bone health [14, 15]. This hypothesis has also been supported by many studies. A meta-analysis showed that Ca intake effectively postpones the decreasing trend in the BMD and reduces the risk of fractures in postmenopausal women [16–19]. However, the effect of Ca on preventing postmenopausal osteoporosis was not consistent in recent data. Reid reported that the effect of calcium supplementation on fractures in healthy older women remained uncertain [20], and some meta-analyses showed that the evidence of the effectiveness of Ca treatment for fractures in postmenopausal women remained limited [21].

Many explanations for the controversial results of meta-analyses of population studies have been proposed, such as different dietary Ca intake levels, national Ca recommendations, vitamin D intake statuses, Ca supplement dosages and durations, and poor long-term compliance. A rat bone loss model was induced by performing OVX and administering a low Ca diet to clarify whether Ca supplementation is beneficial for the prevention of osteoporosis induced by estrogen and Ca deficiencies. Then, different concentrations of calcium carbonate (CaCO_3_) were administered to rats to observe the effects of Ca supplementation on bone loss and explore the possible mechanism.

Metabolomics can be used to collect information on metabolites, including lipids, amino acids, sugars and vitamins, in blood, urine and tissues, and changes in metabolites are sensitive indicators of nutrition and metabolism [22–24]. Metabolomics has been widely used to identify biomarkers and explore the mechanism of osteoporosis [25–27]. Here, the mechanism underlying the effect of Ca on osteoporosis was explored with metabolomics after rats were supplemented with different concentrations of Ca for 13 weeks.

## Methods

### Rat maintenance

CaCO_3_ was obtained from the Harbin Pharmaceutical Group, China (Batch No. 20190227). Forty-eight 7- to 8-week-old female Sprague–Dawley rats with body weights of 250–300 g were purchased from Beijing WeitongLihua Experimental Animal Technology Co. Ltd., license number: SCXK (Peking) 2016-0011. The rats were maintained on a standard 12 h light/12 h dark illumination cycle with water and chow provided ad libitum.

### Experimental design

After 7 days of acclimation, all rats were anesthetized with an intraperitoneal injection of pentobarbital sodium (30 mg/kg BW). All rats underwent either bilateral OVX (n = 36) or sham operation (SO, n = 12). For the sham-operated rats, bilateral abdominal incisions and sutures were performed without oophorectomy [28]. Uterine atrophy (observed during dissection) indicated a successful operation in all OVX rats. After 7 days of convalescence, all rats were assigned to 4 groups and fed a low Ca diet: sham-operated rats fed a low Ca diet (SHAM-LC, n = 12), OVX rats fed a low Ca diet (OVX-LC, n = 12), OVX rats fed a low Ca diet and treated with 750 mg/kg CaCO_3_ (OVX-LC-M, n = 12) or OVX rats fed a low Ca diet and treated with 2800 mg/kg CaCO_3_ (OVX-LC-H, n = 12). The rats in the SHAM-LC and OVX-LC groups were administered distilled water. CaCO_3_ or distilled water was administered to rats by oral gavage for 13 weeks. The experimental protocol was approved by the Animal Ethics Committee of National Institute of Nutrition and Health, Chinese Center for Diseases Control and Prevention.

### Preparation of the low Ca diet

The low Ca diet was prepared according to the “Test method for improving bone mineral density function” inspection and assessment standard for health food issued by the Ministry of Health, People’s Republic of China. The diet was composed of 32% corn starch, 30% sucrose, 23% casein, 5% fiber, 5% corn oil, 3.5% mineral mixture, 1% vitamin mixture, 0.3% DL-methionine, and 0.2% choline tartrate. The following mineral mixture (per kilogram diet) was used: MnSO_4_ 110 mg, CuSO_4_ 0.8 mg, FeSO_4_ 1.2 mg, KI 18.0 mg, ZnSO_4_ 2960 mg, CaHPO_4_ 2890 mg, and MgSO_4_ 12.5 g. The following vitamin mixture was used (per kilogram diet): vitamin A 1.4 × 10^4^ IU, vitamin D 1500 IU, vitamin E 120 mg, vitamin K 3 mg, vitamin B_1_ 12 mg, vitamin B_2_ 20 mg, vitamin B_6_ 12 mg, vitamin B_12_ 0.03 mg, nicotinic acid 60 mg, pantothenic acid 24 mg, folate 6 mg, and biotin 0.54 mg. The Ca content in the low Ca diet was 2.2 g/kg diet after examination.

### Analyses of serum parameters

At the termination of the study, all rats were fasted overnight, and abdominal aortic blood was collected. The levels of Ca, phosphorus (P), glucose (GLU), total cholesterol (TC), triglycerides (TGs), high-density lipoprotein (HDL), low-density lipoprotein (LDL), and nonesterified fatty acids (NEFAs) in serum were examined with an automatic biochemistry analyzer (Hitachi 7600, Japan). The content of 25(OH)D in serum was determined with an ultra-performance liquid chromatography-tandem mass spectrometer (UPLC-MS/MS, Shimadzu 8060, Japan). Serum estradiol (E2) concentrations were assayed with an automatic immune analyzer (DXI800, Beckman, USA). Bone turnover markers (BTMs), including procollagen I N-terminal peptide (PINP), osteocalcin (OC), N-telopeptide of type I collagen (NTX), and β-crosslaps (β-CTX), were detected with rat ELISA kits (Cusabio Biotech Co., China).

### Femoral BMD analysis

The left femur was extracted and examined with a dual-energy X-ray absorptiometry system (DXA, Hologic, USA). The BMD of the whole femur and distal end of femur was analyzed. The placement positions of each femur were consistent. The region of interest (ROI) was defined manually after the scouting scan. The densitometer was calibrated using small animal quality assurance phantoms provided by the manufacturer and performed using established procedures before the series of measurements was collected.

### Histomorphometric analysis of the femur

After fixation with 10% buffered formalin for 5 days, the right femurs were decalcified with 10% EDTA for 3 days. Then, the distal metaphyses of femurs were dehydrated in ethanol, defatted in xylene, embedded in paraffin, and sliced into longitudinal sections (5-μm thick). H&E staining was performed on the sections. The morphology of the rat trabecular bone in the distal femoral metaphyses was observed, and the static parameter of Tb.Ar % within 3 mm under the epiphyseal plate were calculated with Image-Pro Plus 6.0 software.

### Metabolomics analysis

The metabolomics method described in previous studies was used [29, 30]. Serum (100 μL) was mixed with 0.9 mL of 80% methanol containing 0.1% formic acid (FA). After vortexing for 30 s and ultrasonication for 20 min, all samples were frozen for 1 h at − 20 °C for protein precipitation. Then, the samples were centrifuged at 12,000 × g for 10 min at 4 °C, and the supernatant (800 μL) of each sample was collected and transferred to a sample vial. Quality control (QC) samples were prepared by pooling aliquots of all serum samples for the serum metabolomics analysis. Blank samples (80% methanol containing 0.1% formic acid) and QC samples were repeated every ten samples during data acquisition. The sample vials were stored at − 20 °C until detection.

The UPLC-QTOF MS analysis was performed using a UPLC system (ACQUITY UPLC I-Class, Waters) coupled to an electrospray ionization quadruple time-of-flight mass spectrometer (ESI-QTOF MS) (SYNAPT G2-Si HDMS, Waters). A Waters ACQUITY BEH C18 column (1.7 μm; 100 mm × 2.1 mm) was used for LC separation, and the column temperature was maintained at 40 °C. The flow rate was 0.4 mL/min, and the sample injection volume was 10 μL. Mobile phase A was 0.1% FA in water, and mobile phase B was 0.1% FA in ACN. The following linear gradient was set: Initial to 1 min: 10% B, 0–3 min 10% B to 80% B, 3–8 min: 95% B to 95% B, and 8.1–10 min: 10% B.

High-accuracy MS data were recorded using MassLynx 4.1 software. The capillary voltage was 2.5 kV for positive mode and 2 kV for negative mode, whereas the cone voltage was 30 V for both modes. The source temperature was set to 120 °C with a cone gas flow of 50 L/h, and the desolvation temperature was set to 500 °C with a desolvation gas flow of 800 L/h. Leucine-enkephalin (LE) was used as the lock mass, generating a reference ion at m/z 556.2771 in positive mode and m/z 554.2615 in negative mode, which was introduced by a lockspray at a rate of 10 μL/min for data calibration. The MS^E^ data were acquired in continuum mode using ramp collision energy in two scan functions. For low-energy mode, a scan range of 50–1200 Da, scan time of 0.2 s, and collision energy of 6 V were used. For high-energy mode, a scan range of 50–1200 Da, scan time of 0.2 s, and collision energy ramp of 15–45 V were used.

Raw data were imported into the commercial software Progenesis QI (Version 2.4, Waters) for data processing, which included peak selection, peak alignment and acquiring compound-associated information such as the m/z, retention time and intensity. Next, data filtering was performed to delete low-quality data. Ions with a relative standard deviation (RSD) of more than 30% in QC samples were filtered. These filtered ions fluctuated substantially among samples and were not included in further analyses. PLS-DA (partial least squares discriminant analysis) was performed, and VIP (variable importance in projection) was calculated using MetaboAnalyst 4.0 (https://www.metaboanalyst.ca/) [31] and R project software. R project software was also applied in further data processing and statistical analyses. The pathway analysis was performed using MetaboAnalyst 4.0 software. The column graph was drawn using GraphPad Prism 8.0 software.

### Data analysis

All data are presented as the means ± SD. The differences between groups were analyzed using the T-test or one-way ANOVA with SPSS 16.0 software. Differences were considered statistically significant at *p* < 0.05.

## Results

### Effect of CaCO_3_ on body weight

**Fig. 1:**
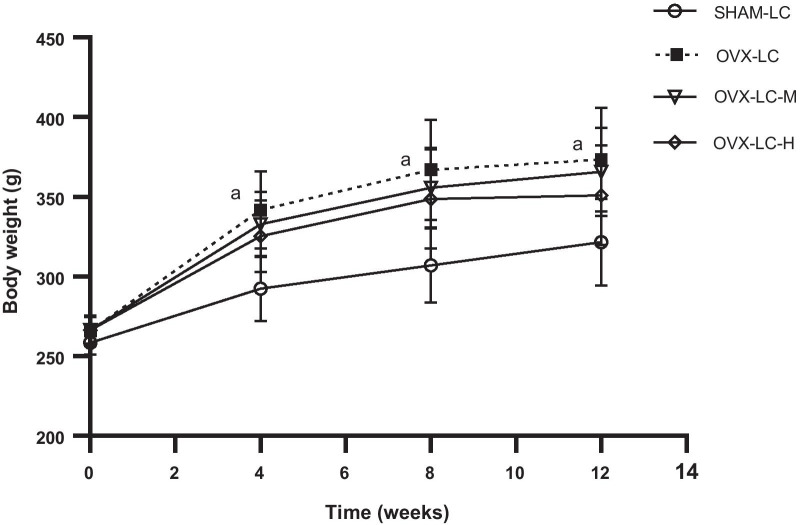
Effect of CaCO_3_ on body weight. **a**: *p* < 0.05 compared with the SHAM-LC group

As shown in Fig. 1, all rats in the four groups had similar initial body weights. The body weight of rats in the OVX-LC group increased significantly after surgery compared to that of the SHAM-LC group (*p* < 0.01). A tendency of reduced body weight after CaCO_3_ treatment was observed, although a significant difference was not observed among the OVX-LC, OVX-LC-M and OVX-LC-H groups. The results indicated that Ca supplementation slightly inhibited the weight gain induced by OVX-LC.

### CaCO_3_ significantly increased the E2 content

**Fig. 2:**
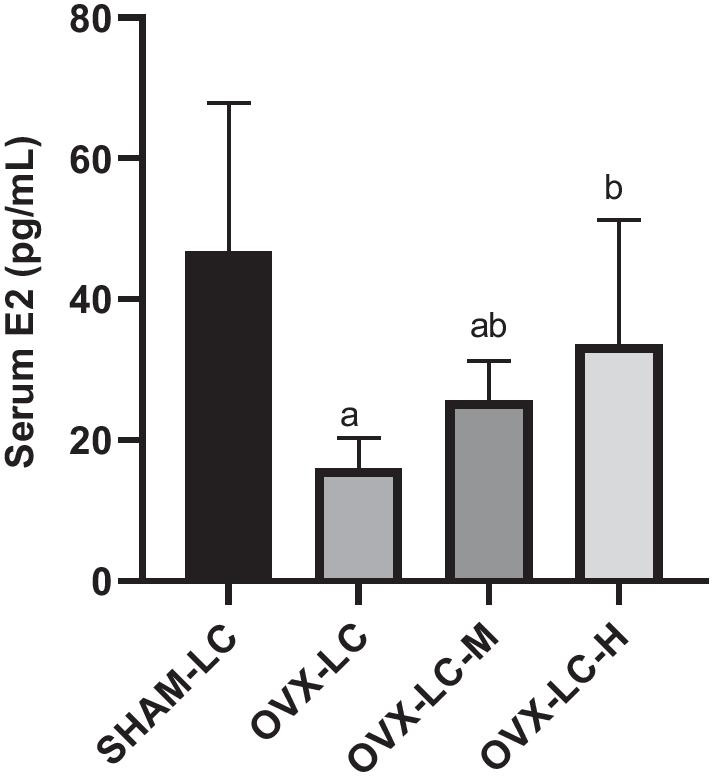
Effect of CaCO_3_ on serum E2 levels. Results are presented as the means ± SD. **a**: *p* < 0.05 compared with the SHAM-LC group. **b**: *p* < 0.05 compared with the OVX-LC group

The induction of postmenopausal conditions by OVX resulted in a dramatic reduction in E2 levels. Compared to the SHAM-LC group, serum E2 levels in the OVX-LC group were reduced significantly (*p* < 0.01) by approximately threefold. Interestingly, serum E2 levels increased significantly in the OVX-LC-M and OVX-LC-H groups compared to the OVX-LC group (*p* < 0.01), and a significant positive dose–response relationship between the dose of CaCO_3_ and serum E2 level was observed (Fig. 2). Ca supplementation increased the E2 concentration in OVX-LC rats, which might be an important factor to alleviate osteoporosis.

### Effect of CaCO_3_ on biochemical parameters

As shown in Table 1, compared to the SHAM-LC group, the serum Ca, P, and 25(OH)D concentrations decreased significantly, and the serum TC, HDL and LDL concentrations increased significantly in the OVX-LC group (*p* < 0.05 or *p* < 0.01). The results indicated that the induction of postmenopausal conditions by OVX led to significant disorders of bone metabolism and lipid metabolism parameters. Compared to the OVX-LC group, the TG and HDL contents in the OVX-LC-H group decreased significantly (*p* < 0.05), and an obvious dose–response relationship was observed among the three groups. No obvious differences were observed in the other parameters examined in serum among the OVX-LC, OVX-LC-M and OVX-LC-H groups. Based on the results, Ca supplementation partially counteracted the disorder of lipid metabolism induced by OVX-LC.

**Table 1:** Effect of CaCO_3_ on serum biochemical parameters

Parameters	SHAM-LC	OVX-LC	OVX-LC-M	OVX-LC-H
Ca (mmol/L)	2.63 ± 0.07	2.52 ± 0.05^a^	2.43 ± 0.22^a^	2.55 ± 0.11
P (mmol/L)	2.54 ± 0.27	2.16 ± 0.31^a^	2.08 ± 0.18^a^	2.10 ± 0.21^a^
GLU (mmol/L)	5.53 ± 0.57	5.73 ± 0.78	6.10 ± 0.90	5.93 ± 1.00
TC (mmol/L)	2.29 ± 0.55	3.32 ± 0.79^a^	3.20 ± 0.40^a^	2.88 ± 0.41^a^
TG (mmol/L)	0.39 ± 0.25	0.63 ± 0.35	0.53 ± 0.31	0.38 ± 0.14^b^
HDL (mmol/L)	1.46 ± 0.32	2.00 ± 0.44^a^	1.87 ± 0.21^a^	1.72 ± 0.23^b^
LDL (mmol/L)	0.62 ± 0.22	1.17 ± 0.43^a^	1.19 ± 0.20^a^	1.01 ± 0.24^a^
NEFA (mmol/L)	0.74 ± 0.31	0.89 ± 0.37	0.82 ± 0.16	0.77 ± 0.21
25(OH)D (ng/mL)	17.48 ± 3.42	12.49 ± 5.06	10.42 ± 4.01	13.17 ± 4.45

Results are presented as the means ± SD, n = 12

^a^*p* < 0.05 compared with the SHAM-LC group

^b^*p* < 0.05 compared with the OVX-LC group

### Effect of CaCO_3_ on BTMs

**Fig. 3:**
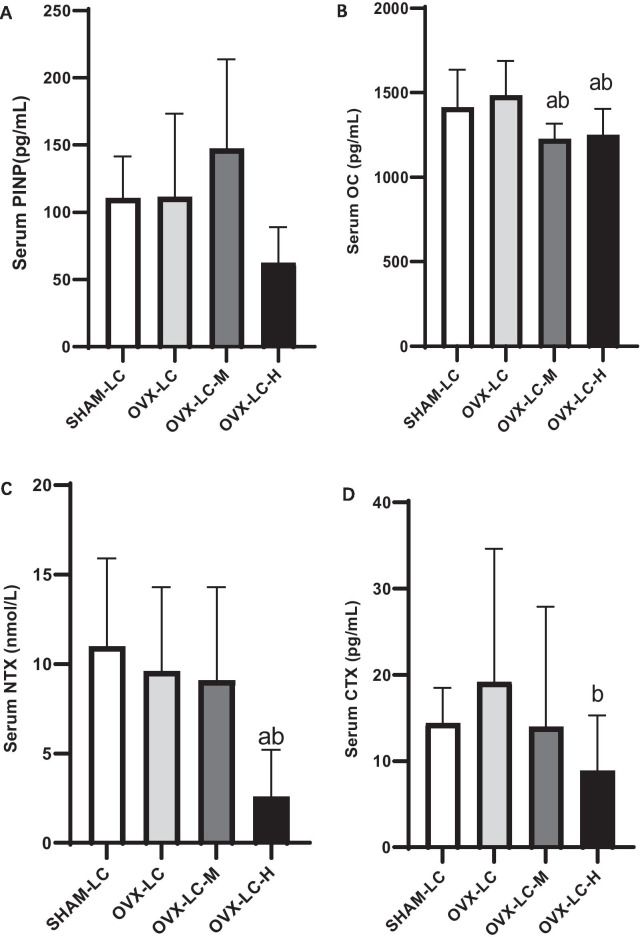
Effect of CaCO_3_ on BTMs. Serum levels of the BTMs PINP (**A**), OC (**B**), NTX (**C**), and CTX (**D**) in all groups were analyzed with ELISAs, n = 12. a: *p* < 0.05 compared with the SHAM-LC group. b: *p* < 0.05 compared with the OVX-LC group

As shown in Fig. 3, compared to the SHAM-LC group, no obvious differences were observed in the four examined BTMs in the OVX-LC group. Compared to the OVX-LC group, the content of OC in the OVX-LC-M and OVX-LC-H groups decreased significantly (*p* < 0.01), and the contents of NTX and β-CTX in the OVX-LC-H group decreased significantly (*p* < 0.05).

### Effect of CaCO_3_ on the BMD of the femur

**Fig. 4:**
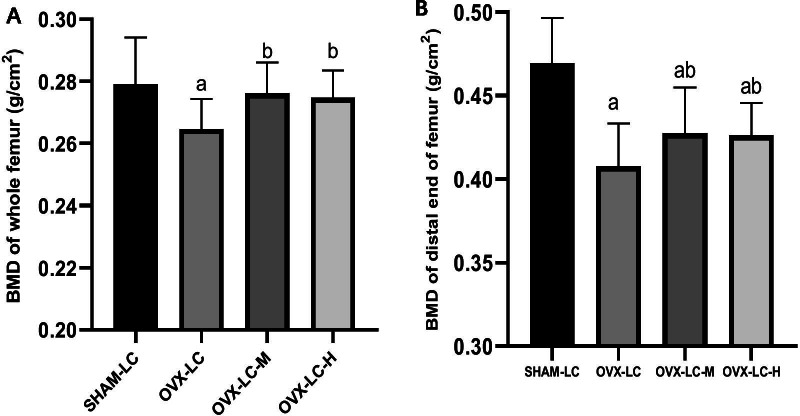
Effect of CaCO_3_ on the BMD of the femur. The BMDs of the whole femur (**A**) and distal end of the femur (**B**) of all groups were analyzed using DXA, n = 12. a: *p* < 0.05 compared with the SHAM-LC group. b: *p* < 0.05 compared with the OVX-LC group

As shown in Fig. 4, compared to the SHAM-LC group, OVX caused a significant decrease in the BMDs of the whole femur and distal end of the femur in the OVX-LC group (*p* < 0.01). The BMDs of the whole femur and distal end of the femur in the OVX-LC-M and OVX-LC-H groups increased significantly compared to those of the OVX-LC group (*p* < 0.05). These results confirmed that the rat bone loss model was successfully established by the OVX operation, while Ca supplementation partially reversed the decrease in BMD in the OVX-LC rats.

### Effect of CaCO_3_ on the histomorphometry of the femur

**Fig. 5:**
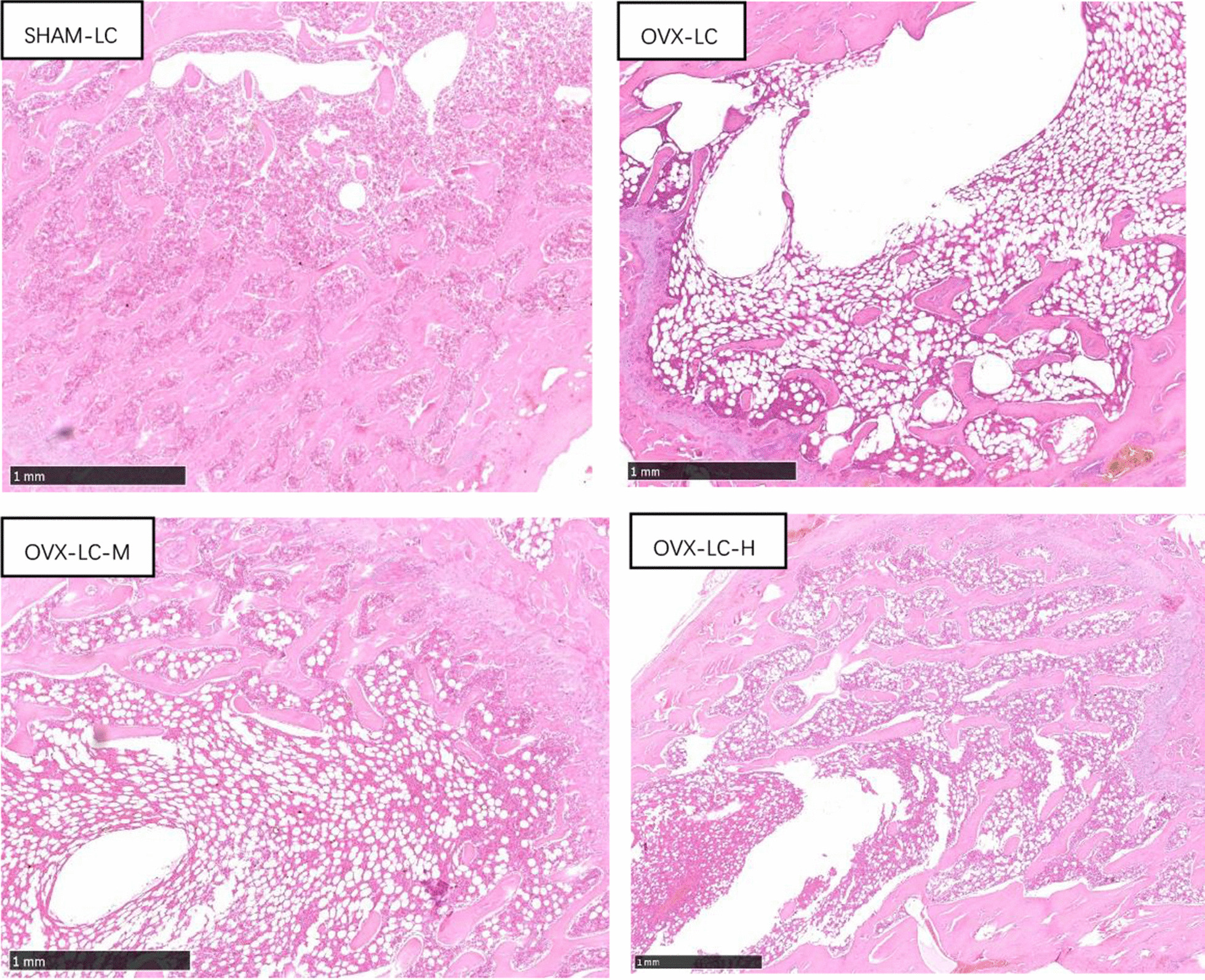
Effect of CaCO_3_ on the histology of the femur. Histological sections (HE.25×) of trabecular bone from rats in different groups were observed. OVX caused an obvious loss of trabecular bone in the OVX-LC group, and the administration of 750 mg/kg or 2800 mg/kg CaCO_3_ significantly rendered the loss of trabecular bone in OVX-LC rats

**Fig. 6:**
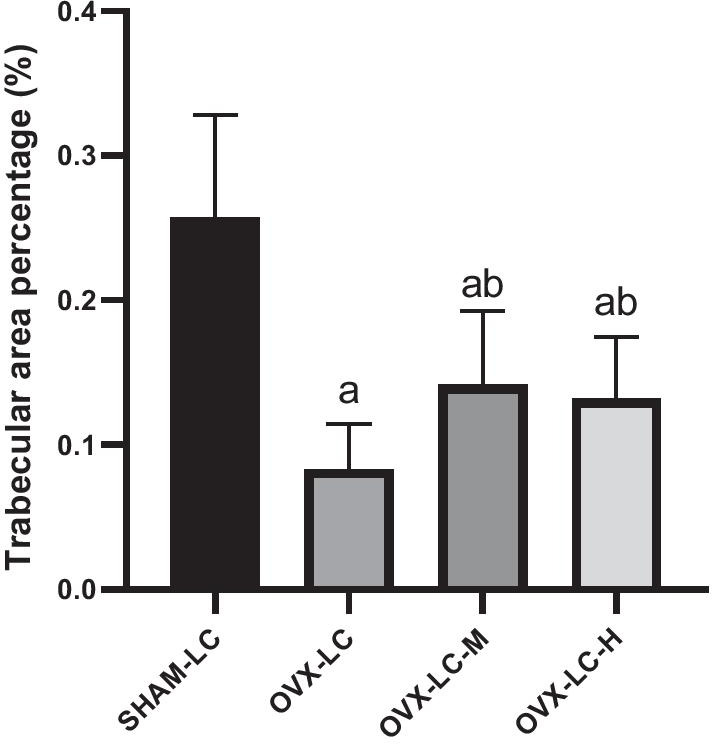
Effect of CaCO_3_ on the histomorphometry of trabecular bone. **a**: *p* < 0.05 compared with the SHAM-LC group. **b**: *p* < 0.05 compared with the OVX-LC group

As shown in Figs. 5 and 6, the structure of trabecular bone in the OVX-LC group became sparse, slender and fractured compared to that of the SHAM-LC group. The changes induced by OVX were accompanied by a significant decrease in the Tb.Ar % (*p* < 0.01). After 13 weeks of CaCO_3_ treatment, improved continuity, integrity and numbers of trabecular bone were observed in the OVX-LC-M and OVX-LC-H groups. Tb.Ar % also increased significantly in the OVX-LC-M and OVX-LC-H groups compared to the OVX-LC group (*p* < 0.05 or *p* < 0.01). Therefore, Ca supplementation improved the structure of the bulk trabecular bone, thereby exerting a protective effect on the bones of OVX-LC rats.

### Effect of CaCO_3_ on serum metabolites

#### PLS-DA model analysis

**Fig. 7:**
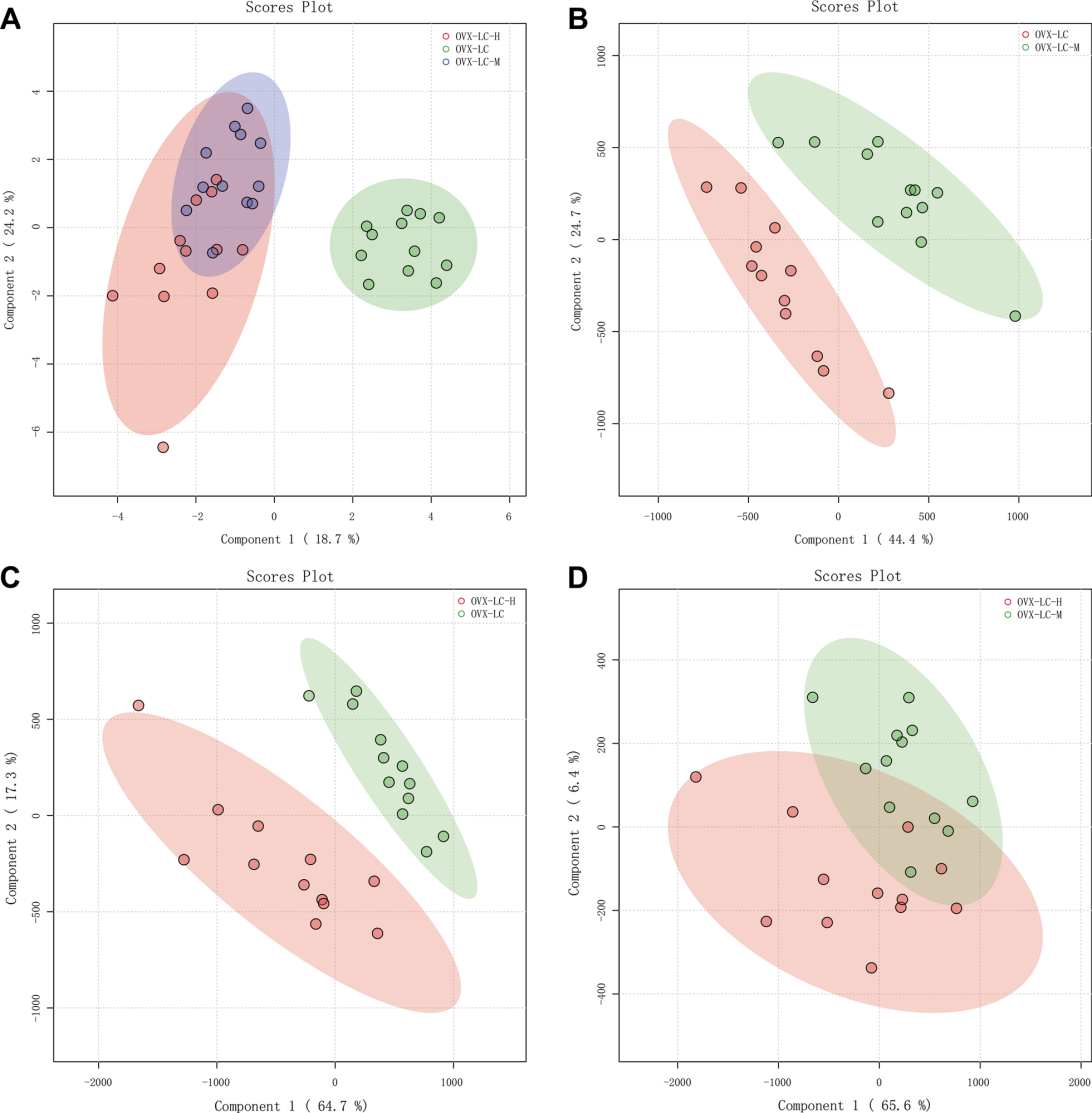
The score figures obtained using the PLS-DA model analysis. **A** Comparisons among the three groups: **B** OVX-LC and OVX-LC-M, **C** OVX-LC and OVX-LC-H, and **D** OVX-LC-M and OVX-LC-H

Nine hundred sixty seven metabolites in rat serum were identified using metabolomics, including glycerophospholipids, triglycerides, diglycerides, ceramides, organic acids, amino acids, fatty acids, and vitamins. According to the PLS-DA multivariate statistical model analysis, obvious differences were discovered on the score graphs among the OVX-LC, OVX-LC-M and OVX-LC-H groups, yet little difference was detected between the OVX-LC-M and OVX-LC-H groups (Fig. 7). The results indicated that calcium supplementation induced changes in metabolites, but no significant difference was observed after the administration of higher concentrations of the Ca supplement.

#### Pathway analysis

**Fig. 8:**
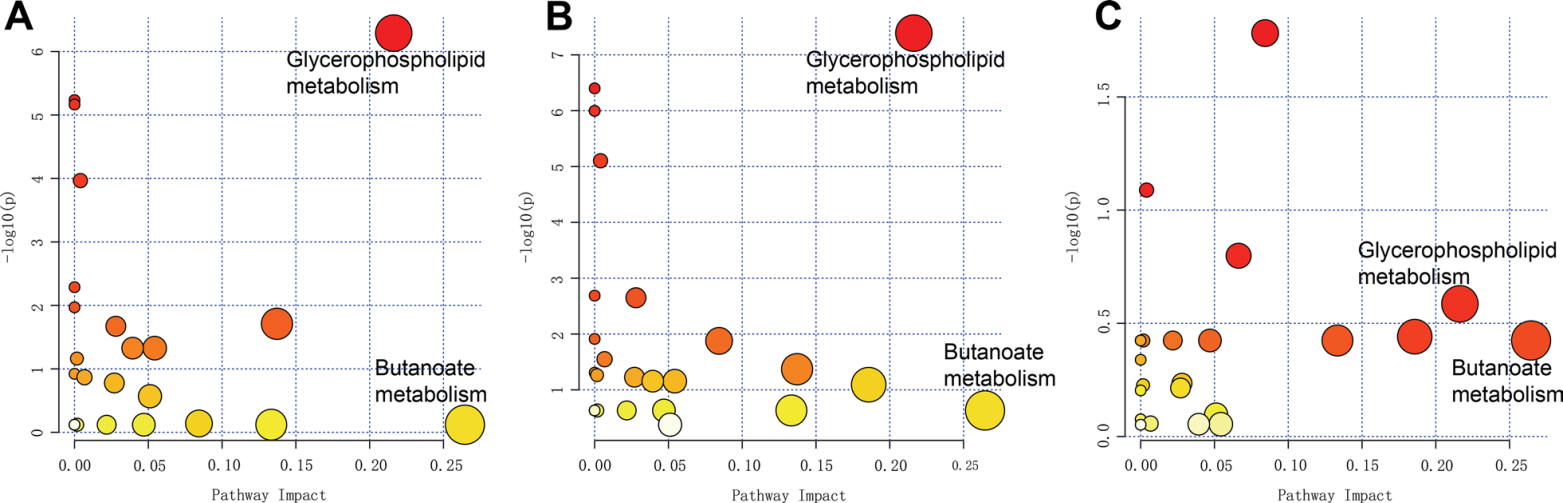
Metabolic pathways that were altered after calcium supplementation in OVX-LC rats. **A** OVX-LC and OVX-LC-M groups, **B** OVX-LC and OVX-LC-H groups, and **C** OVX-L-M and OVX-L-H groups. The horizontal coordinates represent altered pathways, and vertical coordinates represent − log_10_ *p* values, which was 0 farther away from the lower left corner, indicating a larger difference in this metabolic pathway and a greater effect on the pathway. The three metabolic pathways listed above the plots indicated that the glycerophospholipid metabolic pathway showed the greatest difference and effect

We used MetaboAnalyst 4.0 software to analyze and compare the metabolic pathways among the OVX-LC, OVX-LC-M and OVX-LC-H groups, and found that the glycerophospholipid and butanoate metabolic pathways play a major role in the effect of Ca supplementation on OVX rats. However, no significant difference was observed in the butanoate metabolic pathway (*p* values were 0.76, 0.23, and 0.8 for comparisons between the OVX-LC and OVX-LC-M groups, OVX-LC and OVX-LC-H groups, and OVX-LC-M and OVX-LC-H groups, respectively), but a significant difference in the glycerophospholipid metabolic pathway was identified (*p* values were 5.12E−7, 4.08E−8, and 0.26 for comparisons between the OVX-LC and OVX-LC-M groups, OVX-LC and OVX-LC-H groups, and OVX-LC-M and OVX-LC-H groups, respectively). The main differences in glycerophospholipid metabolism were identified in phosphatidylcholines, phosphatidylethanolamines and ceramides (Fig. 8).

#### Cross analysis of differentially altered metabolites

**Fig. 9:**
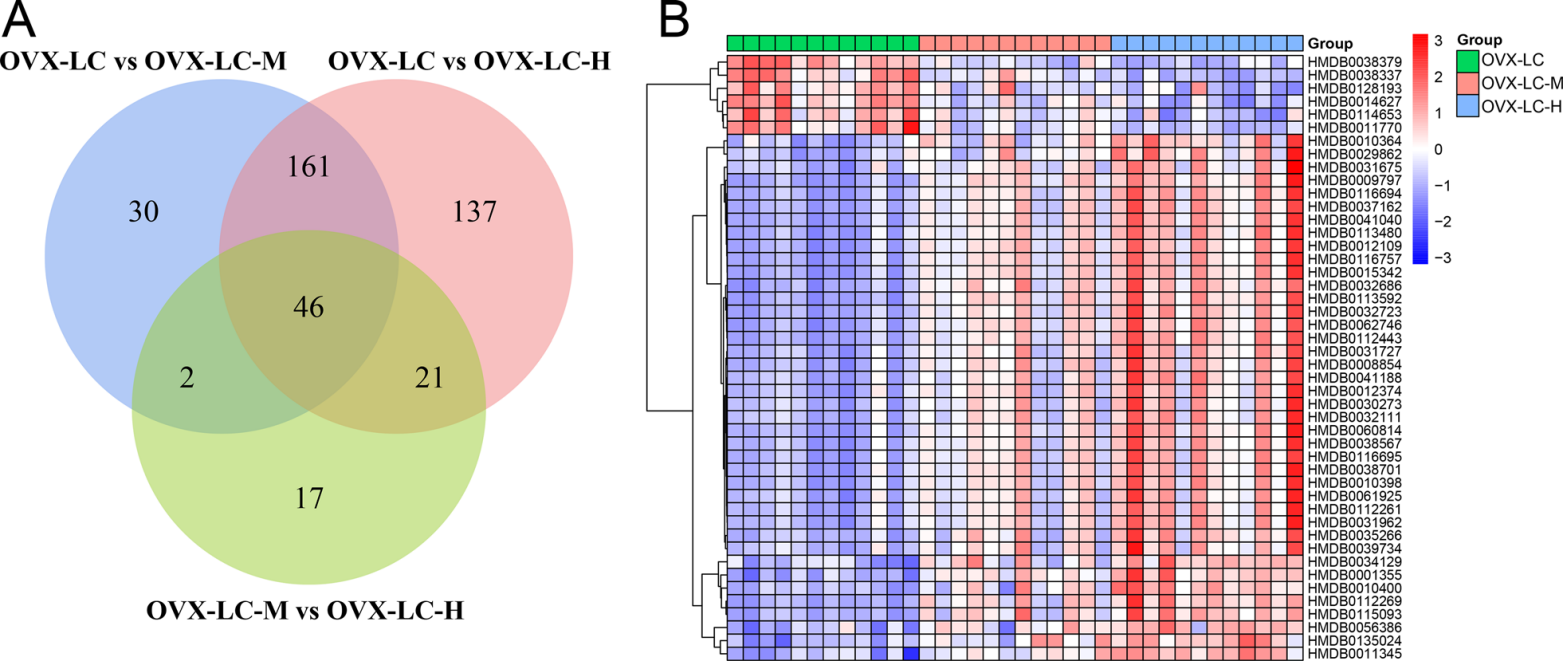
Crossover analysis of differentially altered metabolites between the three groups after calcium supplementation in rats. **A** The Venn diagram clearly shows the similarities and differences in differentially altered metabolites among the three groups, with 46 differentially altered metabolites shared among the three groups. **B** Heatmap analysis of the differentially altered metabolites showing that 6 differentially altered metabolites were downregulated significantly and 40 differentially altered metabolites were upregulated significantly

Through pairwise comparisons, numerous differentially altered metabolites were identified, including 239 metabolites between the OVX-LC and OVX-LC-M groups, 365 metabolites between the OVX-LC and OVX-LC-H groups, 86 metabolites between the OVX-LC-M and OVX-LC-H groups, and 46 metabolites in common among the three groups, as shown in the center of Fig. 9A. The results of the heatmap analysis of the 46 metabolites showed that 6 of them were decreased significantly and 40 of them were increased significantly after Ca supplementation (shown in Fig. 9B).

#### Correlation analysis of Ca and estrogen with differentially altered metabolites

**Fig. 10:**
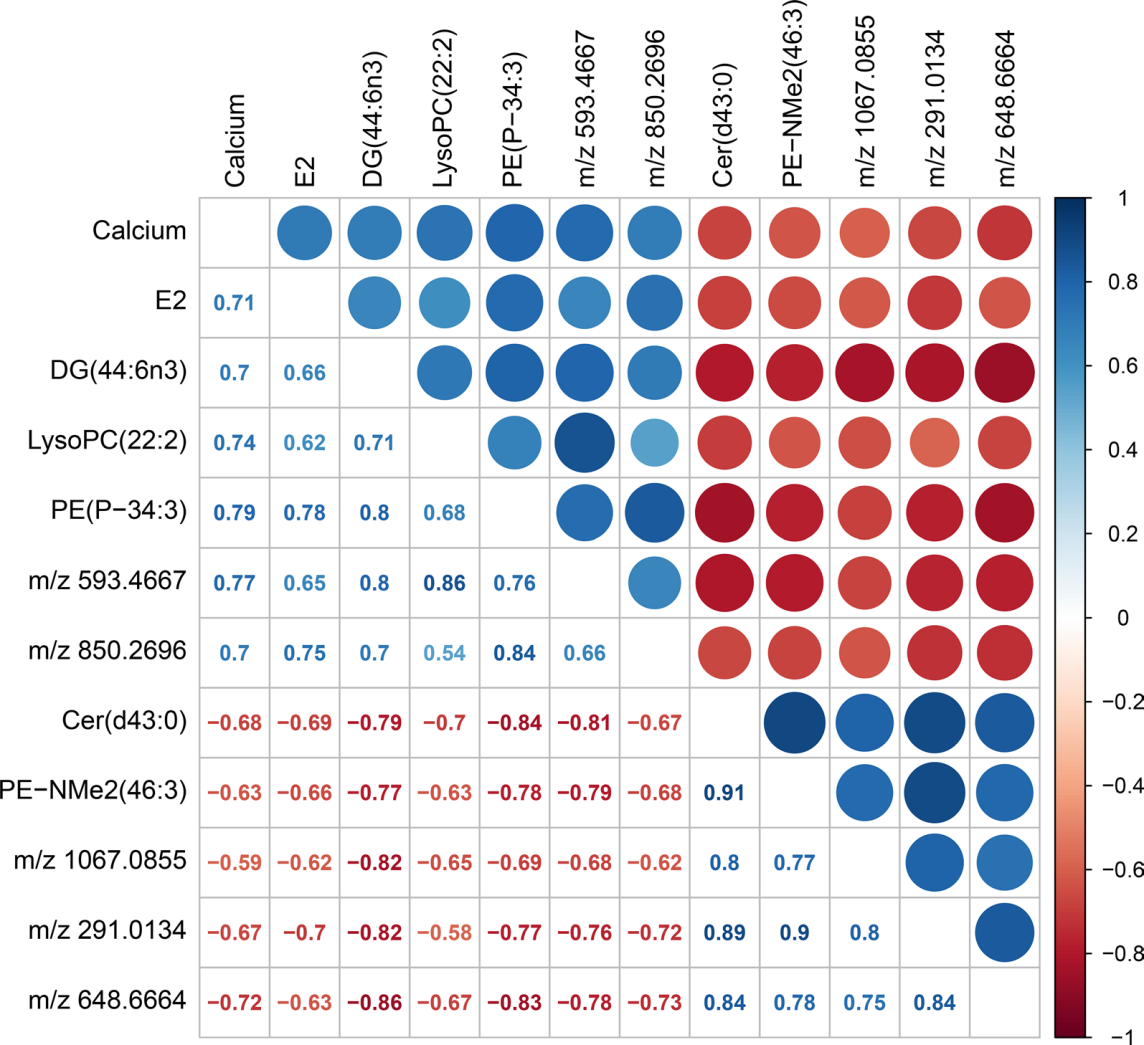
Correlations between calcium and estradiol levels with 46 differentially altered metabolites. The correlation coefficients of 10 of 46 metabolites with calcium were greater than 0.6, with the blue circles and positive values indicating a significant positive correlation and the red circles and negative values indicating a significant negative correlation

The Pearson method was used to analyze the correlations of 46 differentially altered metabolites with Ca and E2 levels. Among the 46 metabolites, 10 were significantly correlated with Ca and E2 levels, and the correlation coefficient was greater than 0.6. The correlation diagram showed that 5 metabolites were positively correlated with Ca supplementation, including DG (44:6n3), LysoPC (22:2) and PE (P-34:3), while the other 5 metabolites were negatively correlated with Ca supplementation, including Cer (d43:0) and PE-NMe2 (46:3), as shown in Fig. 10. This result indicated that Ca supplementation had a close correlation with changes in the levels of some metabolites, especially changes in glycerophospholipid levels.

## Discussion

Ca is an important micronutrient that regulates various physiological activities in the human body, such as the formation of bone and teeth, and maintains the normal function of cells [32]. Sufficient dietary Ca intake is important for maintaining bone health. When Ca intake is insufficient, the organism is forced to increase the process of osteolysis to maintain Ca homeostasis [33]. In the present study, a low Ca diet (22% Ca recommended dose) was used to induce a severe Ca deficiency. Two doses of CaCO_3_ were administered to rats to produce a moderate deficiency and a sufficient Ca intake level to observe the effect of Ca on bone loss induced by OVX-LC.

Estrogen deficiency is the major factor contributing to postmenopausal osteoporosis. Many similarities have been observed between OVX-induced bone loss in rats and postmenopausal osteoporosis in humans. Thus, OVX rats have been widely used as an animal model to study postmenopausal osteoporosis [34, 35]. In the present study, a rat bone loss model was established by OVX-LC. A BMD measurement is considered the standard method for diagnosing osteoporosis in humans, and DXA has been used to measure BMD in rats in many studies [36, 37]. As expected, a significant decrease in the BMD of the femur was observed in the OVX-LC group compared to the SHAM-LC group, which confirmed that estrogen is important for maintaining bone density. The BMD of the whole femur and distal end of the femur in the OVX-LC-M and OVX-LC-H groups increased significantly after 13 weeks of Ca supplementation compared to the values of the OVX-LC group. The changes in the BMD of the femur indicated that Ca supplementation is beneficial to counteract the decrease in bone loss induced by OVX-LC. Gao also reported that a low Ca diet (0.1% calcium) accelerates bone loss in OVX rats [38], consistent with the results of our study.

Deterioration of trabecular bone has been implicated in the incidence of osteoporosis in humans. Thus, an assessment of trabecular bone structure is important to evaluate the quality of the femur in addition to BMD. In this study, a histomorphometric analysis of the femur was performed at the end of the study. The trabecular structure of the femur deteriorated obviously, and the Tb.Ar % decreased significantly compared to that of the SHAM-LC group. The deterioration of trabecular bone was induced by estrogen deficiency, which has also been reported in other studies. After 13 weeks of Ca supplementation, the deterioration of trabecular bone of the femur was ameliorated, and the Tb.Ar % increased significantly in the OVX-LC-M and OVX-LC-H groups. Based on these findings, Ca supplementation contributed to the restoration of trabecular bone.

Decreases in the BMD and deterioration of trabecular bone of the femur were linked to an imbalance in bone remodeling. BTMs are enzymes associated with osteoblasts and osteoclasts [39, 40]. They are intermediate products in the process of bone remodeling. The contents of BTMs in serum or urine reflect changes in bone remodeling before changes in BMD, and BTMs have been suggested as independent risk factors for osteoporotic fractures [41]. Many studies have reported a close association between an increase in the levels some BTMs and an increased risk of hip fracture in the population [42, 43]. For postmenopausal women, the BMD decreases markedly and BTMs increase significantly upon the withdrawal of estrogen [44–46]. In our study, BTMs, including ALP, PINP, OC, β-CTX and NTX, were detected to understand the process of bone formation and bone resorption. Compared to the SHAM-LC group, no significant changes were observed in the OVX-LC group. The changes in BTMs induced by OVX in rats were not consistent with those documented in previous studies [47, 48]. Yan Zhang [49] reported increased urinary deoxypyridinoline (one biomarker of bone resorption) levels at the 4th week after OVX followed by a decrease at the 18th week, and serum ALP (one biomarker of bone formation) levels decreased early and increased late after OVX, indicating that the changes in BTMs after OVX were time-dependent. On the other hand, 2–3-month-old OVX rats were used in the present study. N Patlas reported that rats at 1 and 3 months were more suitable for research on bone histomorphometric parameters [50]. However, continuous physiological bone growth occurs in rats of this age, which might lead to an increase in BTMs. In the present study, serum OC, NTX and β-CTX concentrations in the OVX-LC-H group decreased significantly compared to those in the OVX-LC group, indicating that Ca supplementation slowed the bone remodeling process.

The results of the metabolomics analysis revealed 46 differentially altered metabolites shared among the three groups after Ca supplementation, 6 of which decreased significantly and 40 of which increased significantly. A strong correlation was observed between Ca supplementation and 10 metabolites (correlation coefficient > 0.6), 5 of which had a positive correlation and 5 of which had a negative correlation. These metabolites were mainly glycerophospholipids, including Cer (d43:0), PE-NMe2 (46:3), DG (44:6n3), LysoPC (22:2), and PE (P-34:3). Jiaqi W also reported that one anti-osteoporosis medicine alleviates osteoporosis by regulating glycerophospholipids [51], which is similar to our results showing that glycerophospholipids play an important role in osteoporosis. In addition, studies have also reported that changes in glycerophospholipids are regulated by human hormones, such as estradiol, and glycerophospholipid metabolism is the main potential target pathway of E2 [52].

Estrogen plays an important role in maintaining the balance of bone absorption and bone formation, and estrogen deficiency is known to induce osteoporosis in a variety of animals and in humans [53]. In humans, estrogen replacement appears to be the most efficient method to alleviate postmenopausal osteoporosis regardless of its side effects [54]. In animal studies, estrogen supplementation can also successfully prevent the reduction of BMD in OVX rats [55, 56]. In our study, the serum E2 content in rats increased significantly after Ca supplementation, and an obvious dose–response relationship between the E2 level and Ca content was observed among the OVX-LC, OVX-LC-M and OVX-LC-H groups. Researchers also reported that electroacupuncture and traditional Chinese medicine induce an obvious increase in E2 levels in OVX rats [57, 58]. Since the rats used in our study were OVX rats, E2 was unable to be produced by the ovary. Evidence from recent studies revealed that E2 is synthesized by many nongonadal sites, such as bones, neurons, pancreas, adipose tissue, mesenteric lymph nodes and Peyer’s patches [12, 59–62]. For OVX rats, the capacity of nongonadal sites to synthesize estradiol may be the determining factor in the eventual increase in the BMD.

The results of biochemical analyses in our study showed significant decreases in TG and HDL levels after Ca supplementation. For postmenopausal women, notable changes in blood lipid profiles have been observed upon the withdrawal of estrogen. The results of a meta-analysis showed higher serum HDL and TC concentrations in postmenopausal women with osteoporosis than in normal persons [63]. The results from our study indicated that Ca supplementation alleviated blood lipid disorders in OVX-LC rats.

We are the first to show that Ca supplementation partially restored the estradiol level in OVX-LC rats. According to the results of previous studies, we speculate that the effect of Ca on osteoporosis may be partially mediated by an increase in E2 levels, which then induces changes in metabolites, especially changes in glycerophospholipid and serum lipid levels, and alters the contents of BMTs, eventually increasing the BMD to a relatively higher level to reduce the degree of osteoporosis.

There are two limitations of the study. First, as mentioned in the introduction, the 2010–2012 Chinese National Nutrition and Health Survey showed that the daily Ca intake of Chinese residents was less than half of the recommended intake, leading to Ca deficiency throughout life and not only after menopause. The OVX rats used in the study only mimics postmenopausal Ca and estradiol deficiency. It does not represent the premenopausal Ca deficiency. Second, the rats used in this study are equivalent to teenagers in humans. Old age is not just about menopause but a number of other factors that change during aging. The Ca supplementation proposed in the study may be effective in teenage equivalent OVX rats, however, until proven, it may not produce the same results when tested in old age rats.

## Conclusions

Our results clearly confirm that Ca supplementation is beneficial to reduce bone loss in OVX-LC rats. The main explanation may be that Ca supplementation partially restores the level of estradiol, alters lipid metabolism, induces the production of higher levels of DG (44:6n3), LysoPC (22:2) and PE (P-34:3), and lower levels of Cer (d43:0) and PE-NMe2 (46:3), eventually decreasing the loss of trabecular bone and increasing the BMD of the femur. Originally, researchers proposed that Ca is directly deposited in bone to increase the BMD. A new mechanism by which Ca supplementation potentially increases BMD is by increasing estradiol levels and altering lipid metabolism was proposed. In the future, more studies will be performed to study the effect of Ca on estradiol, and multiomics technology will be used to analyze the upstream and downstream relationship to provide a deeper understanding of the relationship between Ca and osteoporosis.

## Data Availability

The datasets used and/or analyzed during the current study are available from the corresponding author upon reasonable request.

## Abbreviations

ANOVA: Analysis of variance
β-CTX: β-Crosslaps
BMD: Bone mineral density
BTMs: Bone turnover markers
Ca: Calcium
DXA: Dual-energy X-ray absorptiometry system
E2: Estradiol
FA: Formic acid
GLU: Glucose
HDL: High-density lipoprotein
LE: Leucine-encephalin
LDL: Low-density lipoprotein
NEFA: Nonesterified fatty acid
NTX: N-Telopeptide of type I collagen
OC: Osteocalcin
OVX: Ovariectomy
OVX-LC: Ovariectomized rats fed a low calcium diet
OVX-LC-H: Ovariectomized rats fed a low calcium diet and treated with 2800 mg/kg CaCO_3_
OVX-LC-M: Ovariectomized rats fed a low calcium diet and treated with 750 mg/kg CaCO_3_
P: Phosphorus
PINP: Procollagen I N-terminal peptide
PLS-DA: Partial least squares discriminant analysis
QC: Quality control
SD: Standard deviation
SHAM-LC: Sham-operated rats fed a low calcium diet
SO: Sham operation
Tb.Ar %: Trabecular area percentage
TC: Total cholesterol
TG: Triglyceride
UPLC–MS/MS: Ultra-performance liquid chromatography–tandem mass spectrometry
UPLC–QTOF MS: Ultra-performance liquid chromatography–quadrupole time-of-flight mass spectrometry
VIP: Variable importance in projection
